# Spatial and space–time clustering of childhood acute leukaemia in France from 1990 to 2000: a nationwide study

**DOI:** 10.1038/sj.bjc.6602980

**Published:** 2006-02-14

**Authors:** S Bellec, D Hémon, J Rudant, A Goubin, J Clavel

**Affiliations:** 1 INSERM, U754, Villejuif, France; 2 Université Paris-Sud, IFR69, Villejuif, France; 3 French National Registry of Childhood Haematopoetic malignancies, Villejuif, France

**Keywords:** childhood leukaemia, space–time analyses, Moran's I, Potthoff–Whittinghill method, scan statistic, spatial heterogeneity

## Abstract

This study aimed to investigate the spatial and space–time distributions of cases of childhood acute leukaemia (CL) during 1990–2000 over the whole French territory. A global spatial heterogeneity and a spatial autocorrelation were first considered using the methods proposed by Potthoff and Whittinghill, Moran and Rogerson methods. The presence of space–time interaction between the places of residence and the dates of diagnosis was investigated with the Knox's test. Finally, the Kulldorff's statistic permitted to scan the whole territory in search for localised clusters. Two time periods were considered (1990–1994, 1995–2000). Overall, a statistically significant spatial heterogeneity of a very small magnitude was observed in the incidence of CL over 1990–1994, but neither over 1995–2000 nor over the whole time period. Moreover, a significant overdispersion of 5.5% was evidenced for 0–4 year children living in isolated areas with more than 50 inhabitants per km^2^. Cases older than 10 years living in the same area at diagnosis also tended to cluster within 6 months.

The aetiology of childhood leukaemia is still little known with only ionising radiation, certain genetic factors and chemotherapeutic agents established as risk factors. For many years, the detection of clusters and the space–time distribution of cases of childhood leukaemia have been of great interest and widely studied, particularly in Great Britain (Knox, 1964; Draper, 1991; Little, 1999; McNally and Eden, 2004).

Several hypotheses may explain spatial or space–time clustering of childhood leukaemia, such as environmental hazards possibly localised in space and time. In the 1980s a viral hypothesis involving population mixing was proposed after two clusters of childhood leukaemia were detected in isolated areas that had been subject to an unusual population influx. An excess of childhood leukaemia as a rare consequence of an underlying infection might then have resulted from the increased level of contacts between susceptible (more prevalent in rural areas) and infected individuals (Kinlen, 1988). Many studies have supported this hypothesis (for a review see (McNally and Eden, 2004)), though the nature of any underling agent and the way it might be transmitted have not yet been identified.

Two ecological studies on childhood acute leukaemia during 1990–1998 have recently been carried over the whole French territory. No evidence was found of any increased incidence of childhood leukaemia around the 29 nuclear sites (White-Koning *et al*, 2004) but a significant ecological association with the indoor radon concentration was evidenced for acute myeloid leukaemia (Evrard *et al*, 2005). A recent French cohort study also found a positive association between the proportion of newcomers and the incidence of childhood acute leukaemia, particularly in isolated areas with a population density >50 inhabitants km^−2^ (Rudant *et al*, 2005).

The present study, based on the French national registry of childhood haematopoietic malignancies, aimed to investigate the spatial and space–time distributions of cases of childhood acute leukaemia over the whole territory during the period 1990–2000.

## MATERIALS AND METHODS

### Cases

The French national registry of childhood haematopoietic malignancies has registered all cases of acute leukaemia and lymphoma diagnosed from 1990 in children aged up to 14 years old and living in metropolitan France at diagnosis.

All cases of childhood acute leukaemia registered with a date of diagnosis between 1990 and 2000 were included in the present study. The national registry was associated with an estimated 99.2% rate of cases ascertainment (Clavel *et al*, 2004).

### Population and administrative units

Metropolitan France has a total area of 543 965 km^2^ and is divided into 3687 *cantons* and 36 565 *communes*. The last census (1999) indicated a total population of 58.5 million inhabitants, with about 18% of the population aged <15 years.

Since some *commune*s have merged or split, administrative boundaries may vary. For the present study, France was divided into 36 343 areas. Those areas reflect the current French *communes* with the exception of a few mergers, and will therefore be referred to as *communes* throughout. The expected number of cases (E) over 1990–2000 varied from 1 × 10^−5^ to 141.6 with an under-15-year population (P_0–14_) in the range 1–287 636 inhabitants. The 3644 French *cantons* were also considered (E in 0.01–141.6 and P_0–14_ in the range 22–287 636 inhabitants).

The age- and gender-specific populations between the two censuses of 1990 and 1999 were estimated by applying a log–linear diagonal interpolation method that took into account the numbers of births, deaths and migrations each year (White-Koning *et al*, 2004). The main hypothesis underlying this method was the stability of the migration rates between the two censuses. The population estimates for the year 2000 were then equal to those of 1999.

To evaluate the influence of such estimates on the results, two additional scenarios of population growth were implemented. With the first one, the populations over the periods 1990–1994 and 1995–2000 were constant and equal to the population of 1990 and 1999, respectively. In the second one the average population between 1990 and 1999 was applied to each year from 1990 to 2000.

### Statistical methods

Spatial and spatio-temporal methods have first been developed more than 40 years ago (Moran, 1948; Knox, 1964; Naus, 1965; Potthoff and Whittinghill, 1966). Since then some improvements have been made, new approaches have emerged and several methods are now widely used. These methods mainly differ from each other in their objectives. Most of them permit to test for the existence of global heterogeneity, in terms of overdispersion, spatial autocorrelation or space–time interaction, while a few others scan for potential places and periods with higher incidence of disease, with no hypothesis *a priori* (tests for cluster detection).

In the present study, cases were located in space and time according to their c*ommune* or *canton* of residence and date of diagnosis.

### Global spatial heterogeneity

Three methods were considered to test for the existence of a global heterogeneity in the incidence of childhood acute leukaemia in France.

The first one (Potthoff and Whittinghill, 1966) assumes that the number of cases in each area is Poisson distributed under the null hypothesis of no spatial heterogeneity and follows a negative binomial distribution under the alternative hypothesis of overdispersion with a variance to the mean ratio equal to 1+*β*. Based on previous results (Rudant *et al*, 2005), this method was also applied focusing on isolated *communes* with more than 50 inhabitants per km^2^. Isolated areas were defined as non attractive *communes*, in terms of employment, that were included in a unit either rural or urban with less than 5000 inhabitants.

The second method evaluates an autocorrelation index between the incidence rates in the geographical units (Moran, 1948; Elliott and Best, 2000). Two units were considered as neighbouring areas if they are up to *d* kilometres apart. A limit of 40 km was first considered as it insured that none of the areas had no neighbour. To evaluate the stability of the results and to determine the scale at which spatial autocorrelation may occur, three additional values of *d* were considered hereafter (20, 30 and 50 km).

Finally, a global statistic that takes account of both within and across areas variability was considered (Rogerson, 1999). This statistic, based on Tango's index, is a combination of an autocorrelation term and the common *χ*^2^ statistic of goodness-of-fit that compares observed-to-expected regional counts. Neighbouring areas were again defined with limits of 20, 30, 40 and 50 km.

As a result of being computer intensive, Moran's and Rogerson's tests were applied at the *canton* level.

The statistical significance levels based on the one-sided tail probabilities of the null distributions were obtained via Monte Carlo simulations. Under the null hypothesis of no spatial heterogeneity in the incidence rate of childhood acute leukaemia, the total number of observed cases was randomly distributed among the French areas according to a multinomial distribution with parameters proportional to the expected numbers of cases. The statistical significance was then estimated as the proportion of simulations with a statistic greater than or equal to the value observed on the real data. In all, 999 simulations were run.

A normal approximation was also considered to evaluate the statistical significance level with Moran's method (Cliff *et al*, 1973).

### Space–time interaction

The Knox method (Knox, 1964) was used to determine whether the observed number of pairs of cases both close in space and time significantly differed from that expected under the null hypothesis of no space-time interaction. In the present study, the closeness was successively defined by the following spatial and temporal limits: 0, 5, 10, 15, 20, 30 and 50 km; 1, 3, 6, 9 and 12 months. The observed value of the statistic was then compared to its distribution under the null hypothesis. This distribution was obtained by considering 499 permutations of the time of diagnoses, the places of residence being fixed.

### Detection of clusters

The Kulldorff's method permitted to scan the whole territory and the whole time period in search for any particular area and/or time period that may be associated with a higher incidence of disease (Kulldorff and Nagarwalla, 1995). The whole territory was covered by a circular moving window with variable radius and centred on each geographic unit. In the present study, the greatest radius was determined so that the window regrouped up to 10% of the total population. The most likely cluster was then defined as the geographical units included in the window of highest likelihood ratio. A statistical test, under the null hypothesis that the probability of being a case is the same outside and inside the latter window, was then carried out. This method was applied over 1990–2000, 1990–1994 and 1995–2000 separately.

Over 1990–2000, a space–time version of this test was also considered using a cylindrical moving window, the basis and the height of which represented the spatial and the temporal dimensions, respectively. Up to 50% of the whole time-period could have been included in the window.

The significance level was given by 999 Monte Carlo simulations.

### Analyses

Three time periods (1990–2000, 1990–1994, 1995–2000), four age groups (0–14 years, 0–4 years, 5–9 years, 10–14 years) and three groups of diagnosis (AL: acute leukaemia, ALL, acute lymphoblastic leukaemia and AML: acute myeloblastic leukaemia) were considered.

All the analyses were mainly performed with the SAS® and R softwares, and tests for clusters detection were run in SaTScan™ v3.0.5 (Kulldorff and Information Management Services, 2002).

## RESULTS

### Global spatial heterogeneity

#### Spatial overdispersion

The results for the Potthoff–Whittinghill test for spatial heterogeneity are presented in Table 1a. No spatial overdispersion was evidenced, whatever the period, the age group and the group of diagnosis. One may notice, however, a lower statistical significance level for the 0–14 age group for the period 1990–1994, with a 1.2% overdispersion in the incidence of acute leukaemia and particularly acute lymphoblastic leukaemia.

Focusing on isolated *communes*, a statistically significant spatial overdispersion of 1.6% was evidenced in the 0–14 year age group over 1990–1994 (Table 1b). A greater effect (5.5%) was found in isolated *communes* with a population density >50 inhabitants km^−2^, while no overdispersion was observed below this limit. Likewise, a 2.5% overdispersion, although nonsignificant, was found among <4 year children living in isolated *communes* of highest population density. No spatial overdispersion was detected for children aged more than 5 years. No significant overdispersion was found over the whole time period or 1995–2000.

Overall, these results remained quite stable when the alternative scenarios of population estimates were considered (results not shown).

#### Spatial autocorrelation

The analysis of spatial autocorrelation was first conducted considering a limit of proximity of 40 km (Table 2). A significant spatial autocorrelation in the incidence of childhood acute leukaemia, although of a small magnitude (*I*=0.006), was found over the whole period 1990–2000 and 1990–1994. No particular pattern emerged in any of the three age groups. A similar effect was observed with 20 km (*I*=0.006 and *I*=0.009 over 1990–2000 and 1990–1994, respectively), with, however, a statistical significance level >0.05 over 1990–2000.

Results with AML differed slightly as a very small spatial autocorrelation was observed over 1990–1994, but only for children aged <4 years (*I*=0.008 *P*=0.02). No significant autocorrelation was found with ALL (results not shown).

Overall, the statistical thresholds based on Monte Carlo simulations were similar to those obtained with the normal approximation, which illustrated the robustness of the statistic to a possible non-normality (Cliff *et al*, 1973).

All these results also remained quite stable when an autocorrelation estimate with weights inversely proportional to the numbers of neighbours was considered and little variations were observed with the alternative scenarios of population growths (not shown).

#### Overall spatial heterogeneity

Some overall spatial heterogeneity in the incidence of childhood acute leukaemia was evidenced with Rogerson's statistic (Table 3). This result, found only in the first subperiod 1990–1994, was close to the statistical significance for the 0–14 year old children and more marked for the first age group (*P*=0.04). Some spatial heterogeneity in the incidence of acute leukaemia for 0–4 year children was also found when critical limits of 30 or 50 km were considered to define the neighbourhood, while no heterogeneity was observed with 20 km.

No significant heterogeneity was observed either over 1990–2000 or 1995–2000.

A spatial heterogeneity was evidenced with AML, but only for children older than 10 years (*P*=0.04). No spatial heterogeneity was detected in the incidence of ALL (results not shown).

These results remained quite stable when different population estimates were considered (results not shown).

### Space–time interaction

Overall, using Knox's approach, no space-time interaction between the place of residence and the time of diagnosis was found over 1990–2000 (Table 4). However, an interaction seemed to exist for 0–4 year children within critical distances of 30 km and 3 months. Similarly, children aged more than 10 years and living in the same *Commune* at diagnosis tended to cluster within 6 months.

The analyses carried out on the two subperiods 1990–1994 and 1995–2000 revealed two distinct patterns: over the first period a space–time interaction at a small geographical scale (<5 km) and a time limit around 9 months (6–12 months) was evidenced in the last age group, while clustering was restricted to the youngest children over 1995–2000 with a more significant effect within critical limits of 20 km in space and from 9 to 12 months in time (results not shown).

### Detection of clusters

None of the analyses carried out on the French data led to any significant result (Table 5). No particular area of the French territory and/or time period between 1990 and 2000 was thus identified with Kulldorff's method as being associated with a higher incidence of childhood acute leukaemia.

## DISCUSSION

Spatial and space–time clustering of childhood leukaemia has been studied in many countries, and the existence of an infectious agent involved in the aetiology of the disease has become a privileged hypothesis.

A large variety of methods has been considered in several countries and at different periods, which led to various representations of the spatial and spatial–temporal patterns of the childhood leukaemia. Up to now, very few studies have however focused on the spatial heterogeneity in the incidence of childhood acute leukaemia at a large scale (Petridou *et al*, 1997; Alexander *et al*, 1998). The national French registry of childhood haematopoietic malignancies, with a high level of case ascertainment, constitutes a reliable data set, which enables, among others, to carry out spatial and space–time analyses over the whole territory.

The methods used to test for spatial heterogeneity involved multiple testing. The use of several time periods, age groups and parameters is, however, essential as it may give information on the nature of the clustering present and may generate causal hypotheses. The purpose of this study being mainly exploratory, the usual threshold of 0.05 was used even if it may have increased the overall risk of false positive results.

In the present study, the Potthoff–Whittinghill method permitted to evidence a global spatial overdispersion, over the first period only (1990–1994). However, as it was previously observed in the large EUROCLUS study (Alexander *et al*, 1998) this overdispersion was of a weak magnitude (1.2%).

Focusing on isolated *communes*, as defined in a recent French study (Rudant *et al*, 2006), a somewhat stronger overdispersion was observed in areas with a population density >50 inhabitants km^−2^. This observation supports the hypothesis that population density and geographical isolation, possibly combined to a population mixing effect, may play a role in the incidence of childhood acute leukaemia.

The EUROCLUS study evidenced the influence of population density on the incidence of childhood acute leukaemia (Alexander *et al*, 1999). An extra-Poisson variation was actually observed in the areas with <500 inhabitants km^−2^ while the moderately densely populated areas were associated with the highest incidence rates. Similarly, in an analysis conducted in England, Scotland and Wales a spatial heterogeneity attributed to isolated rural areas was detected particularly in the incidence of ALL among young children (Alexander, 1991). Likewise a spatial heterogeneity was found in Greece, particularly in urban and semiurban areas (Petridou *et al*, 1997). Although no overall extra-Poisson variation was evidenced in the incidence of childhood leukaemia and non-Hodgkin's lymphoma in three metropolitan regions of the United States, Muirhead observed an increase in the incidence rate with the population density (Muirhead, 1995). Finally, a study on small units in Hong Kong found a spatial heterogeneity in the incidence of ALL among young children, with a more marked effect in the presence of extreme population mixing (Alexander *et al*, 1997).

In a second step, Moran's statistic permitted to detect a spatial autocorrelation over whole time period and also over 1990–1994, in the 0–14 year age group only. The observed effect was however of a very small magnitude.

The Potthoff–Whittinghill test is the locally most powerful test for the alternative of overdispersion, but its ability to detect the presence of clusters depends on the size, the number and the locations of the clusters (Alexander and Boyle, 1996). This approach does not either take account of any spatial pattern of the deviations between observed and expected values. It is thus impossible to determine whether observed deviations are spatially correlated or localised at random. Moran's coefficient, on the contrary, aims at detecting the presence of spatial autocorrelation among neighbouring areas but does not take account of within area deviations as the weight matrix usually contains null values on its diagonal. Small areas, that are often contiguous, are likely to be associated with extreme standardised incidence ratios (SIRs), even under the null hypothesis, so that some spatial autocorrelation could be observed due to the instability of the SIRs. This issue has already been evoked and some improvements or new methods have been proposed (Oden, 1995; Rogerson, 1999).

Rogerson's statistic is a few changes apart from a combination of the common *χ*^2^ statistic of goodness-of-fit and an autocorrelation term. Both within- and across-areas heterogeneity are thus taken into account. A spatial heterogeneity in the incidence of childhood acute leukaemia was found over the first subperiod but more particularly, and significantly, for the 0–4 year group.

The main drawback of Rogerson's method, which is also a limitation to the use of the Moran's *I*, is the arbitrary choice of a weight matrix. The neighbourhood was here first defined as two areas being up to 40 km apart. To evaluate the stability of the results, the analyses have also been conducted with 20, 30 and 50 km as a critical limit of proximity. The spatial heterogeneity evidenced over 1990–1994 for the first age group was thus ascertained with 30 and 50 km, but disappeared with 20 km. The existence of a small spatial heterogeneity may be limited to a neighbourhood of around 40 km. On the other hand, the geographical scale at which the spatial heterogeneity was investigated with Moran and Rogerson methods could have diluted a spatial heterogeneity at a lower scale.

Some bias could also stem from the use of the loglinear interpolation method to estimate the population at a small geographical level. Nevertheless, the relative stability of the results with the two population growth scenarios (described section Material and Methods) limited the probability that the observed effects could have been the consequence of errors in the population estimates.

Spatio-temporal methods aim to determine whether cases that occur close from one another in space also tend to be close in time. Two different patterns specific to the periods emerged when applying the Knox's method on the French data. Over 1990–1994, some space–time clustering between the places of residence and the times of diagnosis was evidenced in the 10–14 year age group. Cases living 5 km apart actually tended to be diagnosed within a short-time period (from 6 to 12 months).

A different pattern was identified over the second period (1995–2000), as the space–time interaction was specific to the younger age group.

Space–time clustering in the incidence of childhood leukaemia has been extensively studied particularly in Great Britain. A space–time interaction between dates and places of diagnoses of ALL has often been reported within small temporal and spatial distances (Glass *et al*, 1971; Draper, 1991; Gilman and Knox, 1991; Gilman *et al*, 1999; Birch *et al*, 2000; Akhtar *et al*, 2005), especially among young children (Glass *et al*, 1971; Gilman *et al*, 1999; Birch *et al*, 2000). Birch *et al* also evidenced a space–time interaction between the dates of diagnoses and the places of birth, while McNally *et al* (2002) found a significant interaction between both places and dates of birth for the precursor B-cell subtype of ALL.

On the other hand, in a Swedish nationwide study cases of childhood ALL diagnosed after the age of 5 years tended to cluster when they were born in the same place and a few months apart (Gustafsson and Carstensen, 1999, 2000). However, this interaction was not evidenced with places and dates of diagnoses. This difference with our result may be explained by the fact that only one critical distance in space was considered in the Swedish study (0 km) and the older age group was of a wider range (5–14 years old). Based on Cuzick and Edward method, ALL cases aged more than 10 years also seemed to exhibit some space–time clustering in New-Zealand (Dockerty *et al*, 1999). To the authors' knowledge no other study has ever evidenced space–time interaction in the incidence of childhood leukaemia restricted to more than 10-year-old children.

Knox's method is known to be subject to possible bias in case of nonuniform population shifts over the time period (Besag and Newell, 1991; Kulldorff and Hjalmars, 1999) and the probability to detect space–time clustering due to population shifts increases with the number of close pairs observed (Kulldorff and Hjalmars, 1999).

Based on the 1990 and 1999 censuses data, it appeared that 50% of the French *communes* saw their population vary in a proportion >16%. It was also worth noting that more than 5000 pairs of cases were often involved in the observed clustering for the first age group (Table 4). Besides, even if some significant results have been evidenced with particular parameters in this age group, the magnitude of excess was weak and quite homogeneous over the whole range of critical distances as it varied from 0.91 to 1.08. Despite the concordance with the literature, these observations seriously question the space–time clustering found over 1995–2000 among 0–4 year children.

Last, whatever the period and the age group under consideration, no particular cluster of childhood acute leukaemia was evidenced with Kulldorff's test. Similar results were found in Sweden (Hjalmars *et al*, 1996). Over the last few years, the number of localised childhood cancer clusters reported to the health authorities has shown a tendency to increase in several countries, among which France. However, hardly ever has an environmental factor been hold responsible for the observed excess of cases; such clusters were probably due to chance alone (Bellec *et al*, 2005). Likewise, no excess in the incidence of childhood leukaemia was evidenced over 1990–1998 around the 29 French nuclear installations (White-Koning *et al*, 2004). The moving window method has also been applied on a smaller scale but results were specific to some particular areas and less homogeneous (Besag and Newell, 1991; Kulldorff and Nagarwalla, 1995; Akhtar *et al*, 2005).

The main drawback of Kulldorff's test is the arbitrary choice of the maximum population size of the moving window. In the present study, this limit was fixed to 10% of the whole population size so that the area included in the focused window was each time smaller than the ‘outside area’. Despite this limitation, the scan statistic is known to perform well in the detection of hot spots, especially when the shape of the moving window fits that of the cluster (Besag and Newell, 1991; Kulldorff and Nagarwalla, 1995; Akhtar *et al*, 2005). A large recent simulation study also highlighted the fairly good statistical power associated to this test in several situations of hot spots (Kulldorff *et al*, 2003). The methods based on moving windows are, however, poor at detecting some types of clusters such as long and narrow clusters or clusters due to airborne contamination or to a virus transmission (Besag and Newell, 1991; Kulldorff and Nagarwalla, 1995).

Kulldorff's method did not permit to evidence any cluster of childhood acute leukaemia on the French territory. However, because of its weak power to detect noncircular clusters, hot spots may have been missed. Nevertheless, this exploratory study permitted to detect the presence of a global spatial heterogeneity in the incidence of childhood acute leukaemia over 1990–1994, combined with a space–time interaction. Overall of a weak magnitude, this heterogeneity was increased in isolated *communes* with a population density >50 inhabitants km^−2^, particularly among 0- to 4-year-old children. These findings were somewhat compatible with the hypothesis that childhood leukaemia could be a rare consequence of the transmission of a specific infectious agent, particularly in isolated areas subject to unusual population mixing (Kinlen, 1988). In light of our results, it was, however, difficult to determine whether this phenomenon was specific to a particular age group or diagnosis.

Future statistical models should permit to investigate further and better understand these findings, especially the role played by population density and population mixing.

## Figures and Tables

**Table 1:** Potthoff–Whittinghill test for the existence of a spatial heterogeneity in the incidence of childhood acute leukaemia in France 1990–2000. (a) Spatial heterogeneity over the whole territory (36 343 communes) and (b) Spatial heterogeneity in isolated communes in relation to population density

		**Whole period 1990–2000**		**1990–1994**		**1995–2000**	
	**Age (years)**	**No. cases**	***β̂*^a^ (*P*-value^b^)**	**No. cases**	***β̂* (*P*-value)**	**No. cases**	***β̂* (*P*-value)**
*(a) Spatial heterogeneity over the whole territory (36 343 communes)*							
All AL	0–14	4897	0.5% (0.23)	2236	1.2% (0.06)	2261	0.2% (0.38)
	0–4	2471	0.1% (0.40)	1141	0% (0.40)	1330	−0.25% (0.60)
	5–9	1435	−0.5% (0.75)	666	−0.25% (0.62)	769	−0.2% (0.49)
	10–14	991	−0.4% (0.69)	429	−0.2% (0.45)	562	−0.2% (0.56)
							
ALL	0–14	3993	0.35% (0.32)	1831	1.2% (0.07)	2162	−0.1% (0.52)
	0–4	2045	0.05% (0.40)	851	0.3% (0.28)	1094	−0.2% (0.54)
	5–9	1208	−0.5% (0.73)	559	−0.4% (0.77)	649	0.03% (0.34)
	10–14	740	−0.4% (0.67)	321	−0.6% (0.95)	419	−0.3% (0.62)
							
AML	0–14	837	−0.5% (0.74)	374	0.3% (0.18)	463	−0.4% (0.70)
	0–4	395	0.01% (0.32)	179	0.6% (0.07)	216	−0.2% (0.48)
	5–9	205	−0.3% (0.63)	94	−0.1% (0.47)	111	−0.2% (0.74)
	10–14	237	−0.2% (0.47)	101	0.1% (0.13)	136	−0.15% (0.45)
							

		**1990–2000**		**1990–1994**		**1995–2000**	
		**No. cases**	***β̂*^a^ (*P*-value^b^)**	**No. cases**	***β̂* (*P*-value)**	**No. cases**	***β̂* (*P*-value)**
*(b) Spatial heterogeneity in isolated communes in relation to population density*							
							
	*0*–*14*						
	Isolated *communes*	1469	0.1% (0.29)	700	1.6% (0.04)	769	−0.2% (0.41)
	⩽50 inhab/km^2^	554	0.0% (0.28)	239	0.1% (0.27)	315	0.5% (0.14)
	>50 inhab/km^2^	915	0.5% (0.39)	461	5.5% (0.01)	454	−2.3% (0.92)
							
	*0–4*						
	Isolated *communes*	733	0.2 (0.34)	355	0.3% (0.29)	378	−0.3% (0.53)
	⩽50 inhab/km^2^	292	0.1% (0.34)	125	−0.5% (0.50)	167	−0.1% (0.34)
	>50 inhab/km^2^	441	0.5% (0.35)	230	2.5% (0.10)	211	−0.9% (0.64)
							
	*5–9*						
	Isolated *communes*	423	−0.8% (0.86)	196	−4.3% (0.66)	227	−0.5% (0.63)
	⩽50 inhab/km^2^	151	−0.7% (0.64)	64	−0.3% (0.17)	87	−0.4% (0.28)
	>50 inhab/km^2^	272	−1.3% (0.77)	132	−0.8% (0.64)	140	−0.6% (0.57)
							
	*10–4*						
	Isolated *communes*	313	−0.2% (0.55)	149	0.0% (0.34)	164	−0.3% (0.48)
	⩽50 inhab/km^2^	111	−0.3% (0.43)	50	−0.2% (0.11)	61	−0.3% (0.15)
	>50 inhab/km^2^	202	−0.1% (0.49)	99	0.5% (0.32)	103	−0.2% (0.40)

Isolated *communes*: non attractive *communes*, in terms of employment, that are included in a unit with less than 5000 inhabitants (Rudant *et al*, 2005).

a Following (Alexander and Boyle, 1996), the ratio of the variance to the mean of the number of cases in any area was equal to 1+*β*.

b The Statistical significance level was based on the one-sided tail probability of the null distribution (1000 Monte Carlo simulations).

^c^The Statistical significance level was based on the one-sided tail probability of the null distribution (999 Monte Carlo simulations).

**Table 2:** Spatial autocorrelation in the incidence of childhood acute leukaemia in France (1990–2000) – Moran's statistic I (Elliott *et al*, 2000)

	**Whole period 1990–2000**				**1990–1994**				**1995–2000**			
	**No. cases**	**I**	**Stat^a^**	***P*-value^b^**	**No. cases**	**I**	**Stat^a^**	***P*-value**	**No. cases**	**I**	**Stat^a^**	***P*-value**
Age at diagnosis – *d*=40 km												
0–14 years	4873	**0.006**	1.78	**0.04**	2226	**0.006**	1.72	**0.04**	2647	0.002	0.69	0.26
0–4 years	2458	−0.004	−0.97	0.84	1136	−0.001	−0.29	0.63	1322	−0.002	−0.62	0.76
5–9 years	1427	−0.001	−0.10	0.56	662	−0.001	−0.14	0.56	765	0.001	0.24	0.38
10–14 years	988	0.001	0.50	0.28	428	0.004	1.31	0.11	560	−0.002	−0.39	0.65
												
Spatial limit of proximity *d* – Analyses in the whole age group (0–14 years)												
20 km	4873	0.006	0.95	0.15	2226	**0.009**	1.47	**0.05**	2647	−0.001	−0.09	0.54
30 km	4873	0.002	0.47	0.31	2226	0.005	1.10	0.11	2647	−0.002	−0.46	0.68
**40 km**	4873	**0.006**	1.78	**0.04**	2226	**0.006**	1.72	**0.04**	2647	0.002	0.69	0.26
50 km	4873	0.003	1.04	0.16	2226	0.002	0.67	0.26	2647	−0.002	−0.42	0.65

a Normalized statistic [*I*−*E*(*I*)]/*σ*(*I*) with *E*(*I*)=−1/(*n*−1)=−0.0003 (*n*=3644 *cantons*).

b The statistical thresholds were evaluated with the one-sided tail probability of the distribution of I under the null hypothesis (based on 999 Monte Carlo simulations).

Bold highlights the main results, those associated to a *P-*value <5%.

**Table 3:** Overall spatial heterogeneity in the incidence of childhood acute leukaemia in France using Rogerson's statistic (1999)

	**Whole period 1990–2000**			**1990–1994**			**1995–2000**		
	**No. cases**	***R*_H0_ a**	***R*_Obs_^a^ (p^b^)**	**No. cases**	***R*_H0_**	***R*_Obs_ (p)**	**No. cases**	***R*_H0_**	***R*_Obs_ (p)**
Age at diagnosis – *d*=40 km									
0–14 years	4873	0.74	0.90 (0.09)	2226	1.61	2.04 (0.06)	2647	1.36	1.36 (0.31)
0–4 years	2458	1.46	1.60 (0.25)	1136	3.16	**4.05** (**0.04)**	1322	2.71	2.54 (0.63)
5–9 years	1427	2.51	2.21 (0.79)	662	5.42	4.94 (0.71)	765	4.69	4.50 (0.56)
10–14 years	988	3.63	4.14 (0.19)	428	8.39	9.54 (0.20)	560	6.41	6.67 (0.36)
									
Spatial limit of proximity *d* – Analyses in the 0–4 year age group									
20 km	2458	1.47	1.65 (0.10)	1136	3.49	3.56 (0.12)	1322	2.74	2.58 (0.74)
30 km	2458	1.47	1.67 (0.12)	1136	3.17	**3.86** (**0.05)**	1322	2.72	2.59 (0.65)
40 km	2458	1.46	1.60 (0.25)	1136	3.16	**4.05** (**0.04)**	1322	2.71	2.54 (0.63)
50 km	2458	1.45	1.56 (0.32)	1136	3.14	**4.05** (**0.05)**	1322	2.70	2.44 (0.70)

a *R*_H0_ and *R*_Obs_ refer to the value of the statistic expected under the null hypothesis of nonspatial heterogeneity and the observed value, respectively.

b The statistical thresholds were evaluated as the one-sided tail probability of the distribution expected under the null hypothesis (999 Monte Carlo simulations).

Bold highlights the main results, those associated to a *P*-value <5%.

**Table 4:** Space–time interaction between the dates and places of diagnosis of childhood acute leukaemia in France 1990–2000 (Knox, 1964)

		**Time *t* (months)**				
**Age (y)**	**Distance *d* (km)**	**1**	**3**	**6**	**9**	**12**
0–14	0	0.94 (764)	0.95 (1804)	0.99 (3442)	0.99 (4970)	1.00 (6502)
	5	0.98 (1458)	0.98 (3398)	1.00 (6330)	1.00 (9116)	1.00 (11884)
	10	1.00 (4082)	0.99 (9448)	1.00 (17386)	1.00 (25260)	1.00 (32768)
	15	1.01 (7252)	0.99 (16596)	1.00 (30604)	1.00 (44414)	1.00 (57826)
	20	1.01 (10632)	1.00 (24456)	1.00 (44850)	1.01 (65066)	1.01 (84752)
	30	1.01 (16568)	1.00 (38124)	1.00 (69784)	1.00 (101274)	1.01 (132026)
	50	1.00 (25302)	1.00 (58470)	1.00 (107350)	1.00 (155570)	1.00 (202860)
						
0–4	0	1.04 (284)	0.93 (588)	1.00 (1162)	1.00 (1680)	1.00 (2196)
	5	1.04 (476)	1.00 (1052)	1.04 (2012)	1.01 (2828)	1.01 (3680)
	10	1.03 (1238)	1.02 (2814)	1.02 (5174)	1.01 (7410)	1.00 (9604)
	15	1.04 (2144)	1.02 (4860)	1.02 (8950)	1.01 (12774)	1.01 (16632)
	20	1.02 (2954)	1.02 (6866)	1.02 (12572)	1.02 (18190)	1.02 (23682)
	30	1.02 (4520)	**1.02** (10418)*	1.01 (18972)	1.01 (27458)	1.01 (35832)
	50	1.00 (6698)	1.00 (15496)	1.00 (28506)	1.00 (41236)	1.01 (53866)
						
5–9	0	0.81 (46)	0.95 (124)	1.00 (238)	0.95 (328)	1.00 (444)
	5	0.95 (102)	1.00 (248)	0.99 (450)	0.97 (634)	0.98 (828)
	10	0.99 (304)	0.97 (690)	0.98 (1266)	0.98 (1830)	0.98 (2372)
	15	1.01 (542)	1.03 (1280)	0.99 (2256)	1.00 (3288)	1.02 (4316)
	20	1.01 (820)	1.03 (1928)	0.97 (3332)	0.99 (4862)	1.00 (6410)
	30	0.99 (1282)	1.02 (3050)	0.98 (5342)	0.99 (7784)	1.00 (10222)
	50	0.98 (2040)	1.01 (4882)	0.99 (8720)	1.00 (12734)	1.01 (16562)
						
10–14	0	1.00 (20)	1.13 (52)	**1.32** (112)*	1.20 (148)	1.15 (184)
	5	0.85 (42)	1.03 (118)	1.15 (242)	1.12 (342)	1.09 (432)
	10	0.86 (126)	0.92 (312)	0.96 (600)	0.98 (886)	1.00 (1168)
	15	0.92 (250)	0.97 (608)	1.05 (1210)	1.05 (1746)	1.01 (2190)
	20	0.96 (392)	0.98 (924)	1.00 (1734)	1.00 (2520)	0.99 (3226)
	30	0.99 (648)	0.99 (1490)	0.99 (2750)	0.99 (3990)	0.99 (5162)
	50	0.97 (982)	1.00 (2344)	1.00 (4288)	1.00 (6222)	1.00 (8084)

* *P*<0.05.

These figures refer to O/E (O).

O is the observed number of close pairs of cases (up to *d* kilometres and t months apart) among *n*(*n*−1)/2 possible pairs, *n* being the total number of cases in the period and age group under consideration.

*E* is the number of close pairs expected under the hypothesis of no interaction.The statistical thresholds were evaluated as the one-sided tail probability of the distribution of O under the null hypothesis (499 Monte Carlo simulations).

Bold highlights the main results, those associated to a *P*-value <5%.

**Table 5:** Detection of clusters using the Scan statistic of Kulldorff and Nagarwalla (1995) – spatial and space-time analyses

		**1990–2000**				**1990–1994**				**1995–2000**			
		**0–14 years**	**0–4 years**	**5–9 years**	**10–14 years**	**0–14 years**	**0–4 years**	**5–9 years**	**10–14 years**	**0–14 years**	**0–4 years**	**5–9 years**	**10–14 years**
Spatial analyses	*N*_S_	25	88	3	32	512	13	9	6	37	1	1491	2
	*O*_S_	9	13	4	5	44	4	4	2	8	2	65	2
	*E*_S_	1.41	2.89	0.19	0.34	20.64	0.15	0.21	0.01	0.86	0.01	33.69	0.01
	*P*_S_	0.81	0.62	0.92	0.77	0.44	0.61	0.92	0.81	0.30	0.94	0.09	0.92
													
Space-time analyses	*N*_ST_	3	150	1344	19								
	*T*	1992–93	1991–95	1996–99	2000								
	*O*_ST_	5	17	50	3								
	*E*_ST_	0.13	3.67	21.75	0.03								
	*P*_ST_	0.46	0.57	0.29	0.98								

This table gives, for each period and age group, a description of the area associated with the highest likelihood ratio (most likely cluster): the number of *communes* included in the most likely cluster (*N*_S_, *N*_ST_), the number of observed cases (*O*_S_, *O*_ST_), the number of expected cases (*E*_S_, *E*_ST_) and the period associated to the excess in the space–time analyses (*T*).

The spatial moving window was defined so that it contained up to 10% of the whole French population. In space-time analyses, up to 50% of the time period were covered.

The statistical significance levels (*P*_S_, *P*_ST_) were obtained with 999 Monte Carlo simulations.
